# Molecular Identification and Antifungal Susceptibility Patterns of Clinical Dermatophytes Following CLSI and EUCAST Guidelines

**DOI:** 10.3390/jof3020017

**Published:** 2017-03-23

**Authors:** Yubhisha Dabas, Immaculata Xess, Gagandeep Singh, Mragnayani Pandey, Suneeta Meena

**Affiliations:** Department of Microbiology, All India Institute of Medical Sciences, New Delhi 110029, India; yubhi.aiims@gmail.com (Y.D.); drgagandeep@gmail.com (G.S.); miggipandey@gmail.com (M.P.); suneetameena@gmail.com (S.M.)

**Keywords:** dermatophytes, internal transcribed spacer (ITS) sequencing, antifungal susceptibility, CLSI M38-A2, EUCAST E-Def 9.2 revision, India

## Abstract

Dermatophytes are associated with superficial infections in humans worldwide. The aim of the present study was to determine the species distribution and susceptibility patterns of clinical dermatophytes. Samples received for routine mycological processing from 124 suspected cases attending a dermatologic clinic in a tertiary care hospital were included in the study. On direct microscopy, 74.1% (92/124) were positive and 53.2% (66/124) grew on culture. The isolates were comprised of *Trichophyton* *interdigitale* (56%) followed by *Trichophyton* *tonsurans* (25.7%), *Trichophyton* *rubrum* (7.5%), *Trichophyton* *violaceum* (4.5%), *Microsporum* *gypseum* (4.5%), and *Trichophyton* *verrucosum* (1.5%). Conventional mycological identification was concordant with ITS sequencing except for *T.* *mentagrophytes*. High minimum inhibitory concentration (MIC) values (geometric mean, >1 µg/mL) were observed for *T.* *tonsurans* and *T.* *rubrum* to terbinafine and griseofulvin. This study highlights the shift in epidemiology from *T.* *rubrum* to *T.* *interdigitale*. It also raises a concern of high MICs of terbinafine and griseofulvin among our isolates. Surveillance of antifungal susceptibility patterns can provide clinicians with local MIC data that can further aid in guiding better management in relapse cases of dermatomycosis.

## 1. Introduction

Dermatophytes are a group of closely related species that are keratinophilic and morphologically similar. They have the capacity to invade the keratinized tissue (skin, hair, and nails) of humans and other animals to produce an infection, dermatophytosis, commonly referred to as ringworm [1,2]. The universal occurrence of dermatomycosis as estimated by the World Health Organization is about to be 20% [3].

Ringworm is caused by the members of three genera *Microsporum*, *Trichophyton*, and *Epidermophyton*. These keratinophilic pathogenic organisms are also saprophytic in nature [1,2]. *Microsporum* and *Trichophyton* are human and animal pathogens. *Epidermophyton* is only a human pathogen [2].

These infections are not life-threatening, but they cause physical discomfort to the affected persons, which may even lead to a lower self-esteem. Within the past two decades, the incidence of such infections is on the rise, especially in the immunocompromised patient groups including AIDS, diabetes mellitus, cancer and organ transplantation patients, etc. [2]. These are also associated with secondary bacterial infections that may lead to systemic skin infections [4,5].

Over time, a vast range of antifungals has been used to treat dermatophytosis starting with griseofulvin about six decades ago and the first oral imidazole, ketoconazole, about four decades ago [6]. These drugs were followed by other oral azoles—fluconazole, itraconazole, and efinaconazole—and topical allylamines—terbinafine, butanafine, and naftifine [6]. Nowadays, other antifungal agents including luliconazole, amorolfine, and ciclopirox olamine (pyridine) are also popular in clinical practices [7]. The drugs fluconazole, itraconazole, and terbinafine have shown success when used for systemic treatment [8]. Despite the availability of the wide range of antifungals for therapeutic purposes, the failure in treatment has been extensively reported. This may be multifactorial, and the reasons include the extent of the onychomycosis (total onychomycosis, very thick subungual hyperkeratosis and dermatophytoma), causative agents (high MICs of the dermatophyte causing the infection or the presence of non-dermatophytic species, which do not respond to systemic antifungals, such as *Neoscytalidium*, *Scopulariopsis*, and *Fusarium* sp.), and patient comorbidities (immunosuppressed patients, and some drugs may modify antifungal blood levels), inappropriate/insufficient drug administration, discontinuation of therapy, and noncompliance of the patient [4,8,9].

The exact role of drug resistance in treatment failure is not clearly understood. This prospective study was designed to determine the prevalence of different tinea infections and the species distribution with their susceptibility patterns.

## 2. Materials and Methods

This was a purely laboratory-based study including consecutive samples received in the mycology laboratory from 124 patients clinically suspected of dermatophytosis from the dermatology outpatient department of AIIMS, New Delhi from June 2014 to July 2015. Repeat samples from patients were excluded. Ethical clearance from the institute was not required, as the study incorporated the samples sent for routine fungal investigations and the brief clinical history (demographic data, clinical presentation, and site of involvement) incorporated in the analysis was provided on the investigation requisition form sent with the sample. No additional clinical history was collected from the patients, and no follow-up was performed.

### 2.1. Sample Processing

Samples received were subjected to direct microscopic examination using 10% KOH for skin scrapings or hair and 20% KOH for nail samples. For primary isolation, the samples were inoculated in Sabouraud’s dextrose agar (SDA) slopes and were incubated at 25 °C for 30 days before ascribing them as negative for fungi.

### 2.2. Identification of Isolates

Identification of the isolates was done by standard mycological laboratory procedures including morphology on SDA and potato dextrose agar (PDA). Slide culture or microculture was done to study the morphology of microconidia and macroconidia, the nature of the sporulation, the formation of chlamydospores, or the special structures such as spirals, pectinate, the racquet hyphae on corn meal agar (CMA) and PDA. Other special tests were performed where necessary including hair perforation test and growth on rice grain medium. Biochemical tests with urea hydrolysis, 1% peptone agar, and SDA with 5% NaCl were used for species identification.

### 2.3. ITS DNA Sequencing

The DNA was extracted using liquid nitrogen freezing with mortar pestle grinding followed by phenol chloroform extraction [10]. Segments of DNA comprising the internal transcribed region (ITS) were amplified with Primers ITS1 and ITS4 [11]. The samples were amplified by using the following cycling parameters: one initial cycle of 2 min at 94 °C, followed by 35 cycles of 30 s at 94 °C, 50 s at 56 °C, and 2 min at 72 °C, with one final cycle of 7 min at 72 °C. Sequencing reactions were done with 4 μL of a sequencing kit (BigDye Terminator v3.1 cycle sequencing ready reaction; Applied Biosystems, Carlsbad, CA, USA), 1 µM of the primers (ITS1, ITS4), and 3 µL of the PCR product in a final volume of 10 µL. Sequence analysis was performed by comparison of the test nucleotide sequences with the dermatophyte reference nucleotide sequences obtained from the GenBank database (Available online: http://www.ncbi.nih.gov/GenBank/) and were speciated as the reference ITS sequence with a similarity of >99%. The representative sequences obtained were submitted to the GenBank database: *T. interdigitale* (Genbank accession no. KY427899–KY427905); *T. tonsurans* (GenBank accession no. KY427906–KY427910); *T. rubrum* (Genbank accession no. KY427911–KY427912); *M. gypseum* (Genbank accession no. KY427913).

### 2.4. Antifungal Susceptibility Testing

Antifungal susceptibility was performed using the broth microdilution assay according to Clinical Laboratory Standards Institute (CLSI) approved standard M38-A2 and the European Committee on Antimicrobial Susceptibility Testing (EUCAST) (EDef 9.2 Revision) guidelines suggested for molds [12,13]. Quality control isolates (*Candida* *parapsilosis* ATCC 22019 and *Candida* *krusei* ATCC 6258) were included for both methodologies. The antifungal drugs tested were terbinafine, amphotericin B, itraconazole, and griseofulvin (Sigma Chemical Corporation, St. Louis, MO, USA). The medium used was RPMI-1640 with l-glutamine, without bicarbonate, buffered at pH 7.0 with 0.165 M morpholine propane sulfonic acid (MOPS buffer).

The dermatophyte inoculum suspension was prepared using a spectrophotometer to match the optical density with that of 70% transmittance at a 530 nm wavelength. The final inoculum concentration was from 1 × 10^3^ to 3 × 10^3^ CFU/mL. The assays were incubated at 28–30 °C. 

### 2.5. Statistical Analysis

Statistical analysis was done to assess the correlation between the two antifungal susceptibility testing methods used. The on-scale results were included as obtained, and the high off-scale MICs were converted to the next highest concentration to be included in the analysis. Agreement was evaluated by concordance between the MICs obtained by the two different susceptibility testing methods and was defined as a difference of no more than 2 log_2_ dilutions in the MIC values. In addition, to calculate the intraclass correlation coefficients (ICCs) for the MICs, the values were transformed on log_2_ data and were expressed over a maximum value of 1 with a confidence interval of 95% [14]. All statistical analysis was performed with Statistical Package for the Social Sciences software (version 16.0; SPSS S.L., Madrid, Spain).

## 3. Results

**Figure 1:**
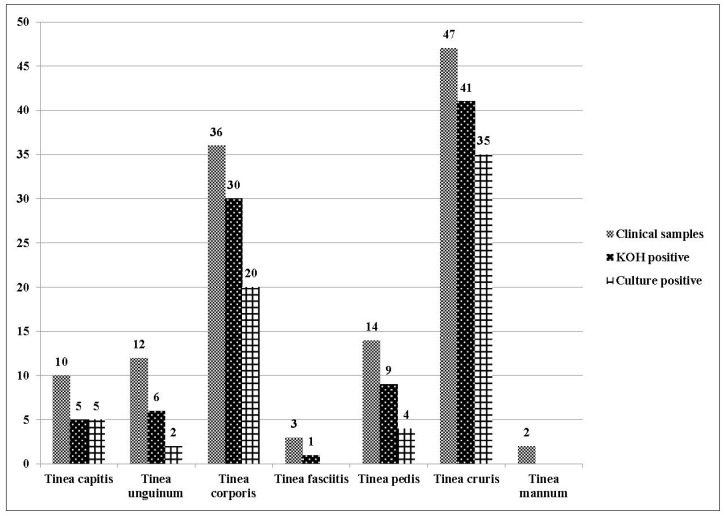
Clinical manifestations, direct microscopy, and culture positivity for all patients enrolled in the study (*n* = 124).

Out of 124 suspected cases of dermatophytosis, 53.2% (66/124) were positive for thin septate hyphae on direct microscopy and grew on culture, whereas 20.9% (26/124) were positive only on direct microscopy with sterile cultures, making a total of 74.1% (92/124) cases in which direct microscopy revealed thin septate hyphae. Clinical manifestation of the patients and the mycological findings are shown in Figure 1.

The mean age of the patients enrolled was 31.2 years. A preponderance of males (79/124, 64%) over females (45/124, 36%) was observed in the selected cohort. The most common clinical manifestation was *Tinea* *cruris* (47/124, 37.9%) followed by *Tinea* *corporis* (29%), and similar results were obtained for the culture positive cases *Tinea* *cruris* (35/66; 53%) followed by *Tinea* *corporis* (20/66; 30.3%). 

### 3.1. Identification of Dermatophytes

**Figure 2:**
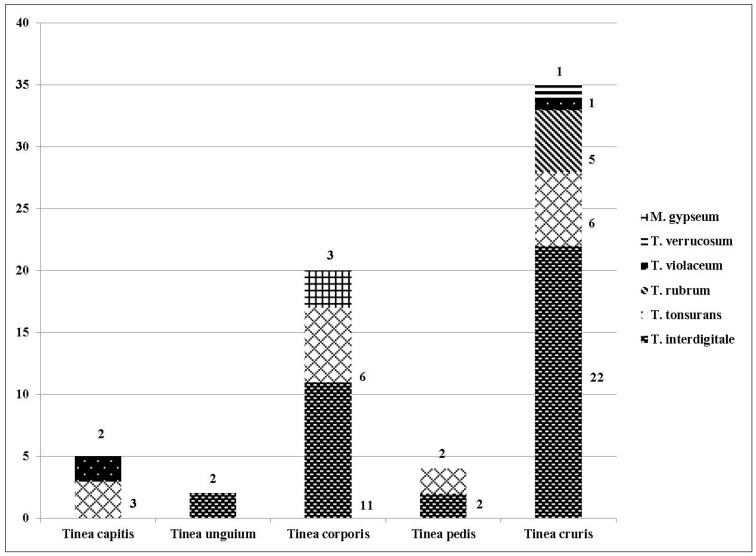
Different species distribution in culture positive patients (*n* = 66).

The most common dermatophytes implicated were *Trichophyton* species in 95.4% (63/66) cases while *Microsporum* species were detected only in (3/66) 4.5% cases. In the present study, no case of dermatophytosis due to *Epidermophyton* species was observed. The most common clinical manifestation with maximum multiple species (5 of 6 different dermatophyte species isolated in the study, 83.3%) involved was *Tinea* *cruris* (35/66, 53.03%) followed by *Tinea* *corporis* (20/66, 30.03%) with the isolation of three different species. *T.* *rubrum* and *T.* *verrucosum* were two species that were recovered only from *Tinea* *cruris* cases, whereas *M.* *gypseum* was isolated only from *Tinea* *corporis* cases. The detailed species distribution in different clinical manifestations is shown in Figure 2.

The conventionally identified *T.* *mentagrophytes* were identified as *T.* *interdigitale* (GenBank accession no. KY427899–KY427905) when subjected to ITS sequencing. The identification for other isolates was concordant for both methods (Out of the total 66 strains, 15 were submitted to the GenBank; *T.* *tonsurans* (GenBank accession no. KY427906–KY427910); *T.* *rubrum* (GenBank accession no. KY427911–KY427912); *M.* *gypseum* (GenBank accession no. KY427913)). According to the species distribution, *T.* *interdigitale* was the predominant organism (56% cases) followed by *T.* *tonsurans* (25.7% cases).

### 3.2. Antifungal Susceptibility Testing

The MIC values of quality control strains were reproducible, fell within the established ranges published by CLSI and EUCAST methodologies. Table 1 summarizes the in vitro susceptibility value ranges of all the isolates to the antifungals tested by the two methodologies followed.

The analysis revealed that high MIC values (geometric mean, >1 µg/mL) were obtained for *T.* *tonsurans* to terbinafine and griseofulvin (EUCAST methodology: 1.379 and 1.995 µg/mL, respectively), and for *T.* *rubrum* to griseofulvin (CLSI and EUCAST methodologies, 3.031 and 5.278 µg/mL, respectively) (Table 1).

Irrespective of the different species, about 50% of our isolates exhibited high MICs (>1 µg/mL) to terbinafine and griseofulvin (Table 2). The detailed statistical analysis between the two methodologies showed high recorded agreement and ICCs between the CLSI and EUCAST results and were within +1 dilution for the antifungals tested, ranging between 83.6% and 98.3% (ICC, 0.73–0.98). 

## 4. Discussion

The present study highlights the clinical manifestations of dermatophytosis, dermatophyte species distribution, and their susceptibility patterns. It was done at a dermatologic clinic in a tertiary care center in Northern India. The climatic conditions (hot and humid environment pertaining to tropical and sub-tropical regions) in India are favorable for the development of superficial fungal infections [15]. The other factors that aid these infections include unhygienic living standards, especially prevalent among the low socio-economic strata, and a high population density especially seen in communities like the ones around construction sites.

The study also reconfirms previous worldwide studies highlighting a high prevalence among males (64%) [16,17]. The mean age of patients was 31.2 years, which is in line as per previously published data showcasing its highest prevalence among the 21–30 age group. This may be due to the general characteristic of this age group as they are more involved in outdoor activities involving physical labor [18,19]. The higher prevalence amongst males may also prove to be an occupational hazard. Many studies indicate *Tinea* *corporis* as the most common presentation followed by *Tinea* *cruris*, but in our study *Tinea* *cruris* was found to be the most common presentation [17,20,21]. 

Our *T.* *mentagrophytes* identifications were based on phenotypic methods, and were found to be incorrect by ITS sequencing as *T.* *interdigitale.* This was not surprising as this has also been reported worldwide [22,23]. For other isolates, the identification results were concordant with conventional and molecular methods. In a study by Li HC et al., it was found that three of the reference strains (BCRC 32066, CBS 160.66, and CBS 361.62) and all clinical isolates when identified by phenotypic methods were found to belong to *T.* *mentagrophytes*, but on ITS sequence analyses, except for a single strain, were identified as *T.* *interdigitale*. The authors also suggested that most of the human isolates of *T.* *mentagrophytes* complex are actually *T.* *interdigitale*, with a few exceptions [24]. These misidentifications are due to morphological identification procedures not keeping pace with phylogenetic studies and nomenclatural changes. Interestingly, by ITS sequencing we found *T.* *interdigitale* (56%) as the predominant species followed by *T.* *tonsurans* (25.7%). *Trichophyton* *rubrum* was seen only in five cases, while it globally causes the maximum tinea infections. This finding is contrary to the observations of others in which an opposite trend has been reported [5,19,24,25].

This change in epidemiology had been previously reported in only three previous studies, by Kaur et al., Adhikari et al., and Yadav et al. from India, where *T.* *interdigitale* (79.2%), *T.* *tonsurans* (44.4%), and *T.* *interdigitale* (61%), respectively, was reported as the most common agents of tinea infections [26,27,28]. Globally, this shift had been reported by Agarwalla et al., Hashemi et al., and Chadeganipour et al., with the most common tinea infections caused by *T.* *interdigitale* [29,30,31].

In this study, we employed the broth microdilution methodologies using two accepted standards, CLSI M38-A2 and EUCAST Edef 9.2, to determine the MICs of antifungal agents for dermatophytes. Globally, the MIC_50_ and MIC_90_ reported for itraconazole, terbinafine, and griseofulvin to dermatophytes were found to be generally low (<1 µg/mL) [32,33,34,35,36,37]. However, there were a few species-specific studies where high MIC values were reported for a few antifungals (>1–32 µg/mL) [36,37]. Our antifungal results showed high MIC values of >2 µg/mL to terbinafine and griseofulvin in about 40% and 20% of our isolates, respectively (Table 2). We also found high MIC_50_ and MIC_90_ (1 µg/mL) for *T.* *tonsurans* and *T.* *rubrum*. However, the number of *T.* *rubrum* was too low (*n* = 5) for a concrete interpretation on the basis of MIC_50_ and MIC_90._ Our observations of high MICs to terbinafine in comparison to itraconazole is in concordance with the recently reported figures by Afshari et al. in 2016 [38]. In another recent study, from India’s northern region, *T.* *mentagrophyte* was identified by phenotypic methods. The species showed low MICs to itraconazole and ketoconazole in comparison to terbinafine (MIC_50_: 0.125 µg/mL for itraconazole, 0.0625 µg/mL for ketoconazole, and 0.5 µg/mL for terbinafine) [4], whereas from the eastern region of India, low MICs to terbinafine in comparison with griseofulvin and itraconazole were reported [34]. To summarize, our data is not in agreement with previously published studies, indicating low MICs to terbinafine [32,33,34,35,36,39]. The authors clarify that the clinical significance of these high MICs is unclear, as patient outcomes were not followed up, and there is a lack of studies in general correlating dermatophyte antifungal MICs with treatment outcomes.

Although breakpoints needed to analyze this data for practical clinical application are not available, this data can aid in understanding local susceptibility patterns. The dissimilarity of our results can be due to species-specific susceptibility against antifungal drugs or/and inter-laboratory and inter-method variations.

The results for amphotericin B were also included in the study for the likely benefit they may provide for isolates refractory to the standard treatment for the dermatophytic infections. The amphotericin B gel has shown promising results in refractory cases of cutaneous fungal infections and non-dermatophyte mold onychomycosis [40,41].

We performed antifungal susceptibility tests on our isolates with conidia (classical inoculum preparation) instead of using a modified protocol of fragmented mycelium inoculum as our isolates had sporulated nicely. However, our results show an 83.6–98.3% agreement between the two methodologies, which is in accordance with those reported in a recent study by Risslegger et al., where they found an 88.9–100% agreement between the modified EUCAST (fragmented mycelium inoculum) and classical CLSI methodology [42]. Since we found a high recorded agreement between the two methodologies and ICCs in this study, a fair comparison with other studies conducted using either of the two standard methods is presented.

## 5. Conclusions

The present study provides useful insights on the reliability of the conventional methods for the identification of dermatophytes. It also provides useful information regarding antifungal susceptibility pattern of dermatophytes and raises important concerns regarding high MIC values of terbinafine and griseofulvin in our isolates. Tinea is not considered a life threatening condition, but it certainly leads to personal discomfort, and antifungal treatment regimens can last for a fairly long duration (3–6 months). To prevent the unnecessary usage of toxic drugs, regular surveillance of antifungal susceptibility patterns in patients should be carried out in their long-term interest.

## Figures and Tables

**Table 1:** MIC value range with geometric mean, mode, and MIC_50_ and MIC_90_ values for the different dermatophytic species by the CLSI M38-A2 and EUCAST methodologies.

Antifungals, Dermatophyte Species, and the Methodologies Followed	MIC Distribution (μg/mL) (No. of Isolates)												
	0.03	0.06	0.125	0.25	0.5	1	2	4	8	16	^+^GM	MIC_50_	MIC_90_
**Itraconazole**													
*T. interdigitale* (*n* = 37)													
CLSI	21	13	3	0	0	0	0	0	0	0	0.042	0.03	0.06
EUCAST	7	6	11	0	3	0	0	0	0	0	0.077	0.06	0.125
*T. tonsurans* (*n* = 17)													
CLSI	15	1	1	0	0	0	0	0	0	0	0.033	0.03	0.06
EUCAST	0	15	1	1	0	0	0	0	0	0	0.07	0.06	0.125
*T. rubrum* (*n* = 5)													
CLSI	3	2	0	0	0	0	0	0	0	0	0.039	0.03	0.06
EUCAST	1	2	2	0	0	0	0	0	0	0	0.07	0.06	0.125
*T. violaceum* (*n* = 3)													
CLSI	2	1	0	0	0	0	0	0	0	0	0.037	0.03	0.06
EUCAST	2	1	0	0	0	0	0	0	0	0	0.037	0.03	0.06
*M. gypseum* (*n* = 3)													
CLSI	2	1	0	0	0	0	0	0	0	0	0.037	0.03	0.06
EUCAST	2	1	0	0	0	0	0	0	0	0	0.037	0.03	0.06
*T. verrucosum* (*n* = 1)													
CLSI	1	0	0	0	0	0	0	0	0	0	*NA	*NA	*NA
EUCAST	1	0	0	0	0	0	0	0	0	0	*NA	*NA	*NA
**Terbinafine**													
*T. interdigitale* (*n* = 37)													
CLSI	3	2	5	5	14	5	0	0	2	1	0.375	0.5	1
EUCAST	0	5	0	4	6	15	4	0	2	1	0.683	1	2
*T. tonsurans* (*n* = 17)													
CLSI	3	0	0	2	2	1	4	2	2	1	0.878	2	8
EUCAST	0	2	1	0	2	2	4	2	3	1	1.379	2	8
*T. rubrum* (*n* = 5)													
CLSI	1	0	0	0	2	0	0	0	2	0	0.863	0.5	8
EUCAST	0	1	0	0	2	0	0	0	2	0	0.991	0.5	8
*T. violaceum* (*n* = 3)													
CLSI	2	0	1	0	0	0	0	0	0	0	0.048	0.03	0.125
EUCAST	0	2	1	0	0	0	0	0	0	0	0.076	0.06	0.125
*M. gypseum* (*n* = 3)													
CLSI	2	0	1	0	0	0	0	0	0	0	0.048	0.03	0.125
EUCAST	0	2	1	0	0	0	0	0	0	0	0.076	0.06	0.125
*T. verrucosum* (*n* = 1)													
CLSI	0	0	0	0	0	0	0	0	1	0	*NA	*NA	*NA
EUCAST	0	0	0	0	0	0	0	0	0	1	*NA	*NA	*NA
**Griseofulvin**													
*T. interdigitale* (*n* = 37)													
CLSI	3	2	5	5	14	5	0	0	2	1	0.375	0.5	1
EUCAST	0	5	0	5	11	13	0	0	0	3	0.577	0.5	1
*T. tonsurans* (*n* = 17)													
CLSI	1	2	0	0	4	3	5	1	0	1	0.777	1	4
EUCAST	0	1	1	1	0	2	4	3	4	1	1.995	2	8
*T. rubrum* (*n* = 5)													
CLSI	0	0	0	0	0	1	1	2	1	0	3.031	4	8
EUCAST	0	0	0	0	0	0	1	1	3	0	5.278	8	8
*T. violaceum* (*n* = 3)													
CLSI	2	1	0	0	0	0	0	0	0	0	0.037	0.03	0.06
EUCAST	2	1	0	0	0	0	0	0	0	0	0.037	0.03	0.06
*M. gypseum* (*n* = 3)													
CLSI	0	2	1	0	0	0	0	0	0	0	0.076	0.06	0.125
EUCAST	0	2	1	0	0	0	0	0	0	0	0.076	0.06	0.125
*T. verrucosum* (*n* = 1)													
CLSI	0	0	0	0	0	1	0	0	0	0	*NA	*NA	*NA
EUCAST	0	0	0	0	0	0	1	0	0	0	*NA	*NA	*NA
**Amphotericin B**													
*T. interdigitale* (*n* = 37)													
CLSI	13	4	18	2	0	0	0	0	0	0	0.072	0.125	0.125
EUCAST	13	4	18	2	0	0	0	0	0	0	0.072	0.125	0.125
*T. tonsurans* (*n* = 17)													
CLSI	4	3	5	0	3	1	1	0	0	0	0.133	0.125	1
EUCAST	0	7	5	0	0	4	1	0	0	0	0.177	0.125	1
*T. rubrum* (*n* = 5)													
CLSI	1	1	0	2	0	1	0	0	0	0	0.162	0.25	1
EUCAST	0	2	0	1	1	0	1	0	0	0	0.245	0.25	2
*T. violaceum* (*n* = 3)													
CLSI	0	0	3	0	0	0	0	0	0	0	0.125	0.125	0.125
EUCAST	0	0	2	1	0	0	0	0	0	0	0.157	0.125	0.25
*M. gypseum* (*n* = 3)													
CLSI	0	0	0	3	0	0	0	0	0	0	0.25	0.25	0.25
EUCAST	0	0	0	3	0	0	0	0	0	0	0.25	0.25	0.25
*T. verrucosum* (*n* = 1)													
CLSI	0	0	0	0	0	0	1	0	0	0	*NA	*NA	*NA
EUCAST	0	0	0	0	0	0	1	0	0	0	*NA	*NA	*NA

(Note: the underlined values denote the modal MICs; *NA: not applicable; ^+^GM: geometric mean).

**Table 2:** Susceptibilities of dermatophytes to various antifungals and the concordance and intraclass coefficients (ICC) between CLSI M38-A2 and EUCAST guidelines.

Antifungal Agents	CLSI (%) (*n* = 66)			EUCAST (%) (*n* = 66)			Concordance	ICC (95% CI)
	<0.5 µg/mL	0.5–1 µg/mL	≥2 µg/mL	<0.5 µg/mL	0.5–1 µg/mL	≥2 µg/mL		
Itraconazole	100	0	0	93.9	6	0	83.6	0.734 (0.038–0.895)
Terbinafine	40.9	36.3	22.7	28.8	40.9	30.3	98.3	0.968 (0.396–0.991)
Griseofulvin	36.3	42.4	21.2	28.8	39.4	31.8	96.7	0.957 (0.501–0.987)
Amphotericin B	89.4	7.6	3	87.9	7.6	4.5	97.2	0.982 (0.965–0.99)
